# Linezolid-Resistant Enterococcus faecalis of Chicken Origin Harbored Chromosome-Borne *optrA* and Plasmid-Borne *cfr*, *cfr*(D), and *poxtA2* Genes

**DOI:** 10.1128/spectrum.02741-22

**Published:** 2023-03-30

**Authors:** Biao Tang, Chenhui Zou, Stefan Schwarz, Chunyan Xu, Wenbo Hao, Xiao-Mei Yan, Yuting Huang, Juan Ni, Hua Yang, Xiang-Dang Du, Xinxin Shan

**Affiliations:** a State Key Laboratory for Managing Biotic and Chemical Threats to the Quality and Safety of Agro-Products, Institute of Agro-product Safety and Nutrition, Zhejiang Academy of Agricultural Sciences, Hangzhou, China; b International Joint Research Center of National Animal Immunology, College of Veterinary Medicine, Henan Agricultural University, Zhengzhou, China; c Institute of Microbiology and Epizootics, Centre for Infection Medicine, Department of Veterinary Medicine, Freie Universität Berlin, Berlin, Germany; d Veterinary Centre for Resistance Research (TZR), Freie Universität Berlin, Berlin, Germany; e State Key Laboratory of Infectious Disease Prevention and Control, Collaborative Innovation Center for Diagnosis and Treatment of Infectious Diseases, National Institute for Communicable Disease Control and Prevention, Chinese Center for Disease Control and Prevention, Beijing, China; University of California, Davis

**Keywords:** *Enterococcus faecali*s, linezolid, resistance, mobile genetic element, horizontal transfer, conjugation, enterococcus, antimicrobial resistance, oxazolidinones, plasmid-mediated resistance

## Abstract

The aim of this study was to investigate the transferability of acquired linezolid resistance genes and associated mobile genetic elements in an Enterococcus faecalis isolate QZ076, cocarrying *optrA*, *cfr*, *cfr*(D), and *poxtA2* genes. MICs were determined by broth microdilution. Whole-genome sequencing (WGS) was performed using the Illumina and Nanopore platforms. The transfer of linezolid resistance genes was investigated by conjugation, using E. faecalis JH2-2 and clinical methicillin-resistant Staphylococcus aureus (MRSA) 109 as recipients. E. faecalis QZ076 harbors four plasmids, designated pQZ076-1 to pQZ076-4, with *optrA* located in the chromosomal DNA. The gene *cfr* was located on a novel pseudocompound transposon, designated Tn*7515*, integrated into the 65,961-bp pCF10-like pheromone-responsive conjugative plasmid pQZ076-1. Tn*7515* generated 8-bp direct target duplications (5′-GATACGTA-3′). The genes *cfr*(D) and *poxtA2* were colocated on the 16,397-bp mobilizable broad-host-range Inc18 plasmid pQZ076-4. The *cfr*-carrying plasmid pQZ076-1 could transfer from E. faecalis QZ076 to E. faecalis JH2-2, along with the *cfr*(D)- and *poxtA2*-cocarrying plasmid pQZ076-4, conferring the corresponding resistant phenotype to the recipient. Moreover, pQZ076-4 could also transfer to MRSA 109. To the best of our knowledge, this study presented the first report of four acquired linezolid resistance genes [*optrA*, *cfr*, *cfr*(D), and *poxtA2*] being simultaneously present in the same E. faecalis isolate. The location of the *cfr* gene on a pseudocompound transposon in a pheromone-responsive conjugative plasmid will accelerate its rapid dissemination. In addition, the *cfr*-carrying pheromone-responsive conjugative plasmid in E. faecalis was also able to mobilize the interspecies transfer of the *cfr*(D)- and *poxtA2*-cocarrying plasmid between enterococci and staphylococci.

**IMPORTANCE** In this study, the simultaneous occurrence of four acquired oxazolidinone resistance genes [*optrA*, *cfr*, *cfr*(D), and *poxtA2*] was identified in an E. faecalis isolate of chicken origin. The association of the *cfr* gene with a novel pseudocompound transposon Tn*7515* integrated into a pCF10-like pheromone-responsive conjugative plasmid will accelerate its dissemination. Moreover, the location of the resistance genes *cfr*(D) and *poxtA2* on a mobilizable broad-host-range Inc18 family plasmid represents the basis for their intra- and interspecies dissemination with the aid of a conjugative plasmid and further accelerates the spreading of acquired oxazolidinone resistance genes, such as *cfr*, *cfr*(D), and *poxtA2*, among Gram-positive pathogens.

## INTRODUCTION

Enterococci are considered commensal organisms but also as human opportunistic pathogens. The species Enterococcus faecalis and Enterococcus faecium are especially known to cause urinary tract, soft tissue, and device-associated infections. Their intrinsic resistance to several common antimicrobial agents and their ability to acquire new antimicrobial resistance genes (ARGs) increase the treatment cost of infections caused by them and increase the risk of treatment failure and death (1). In addition, enterococci are also inhabitants of the intestinal tract of food-producing animals and, as a consequence, are also present in livestock manure. Livestock manure is commonly used as organic fertilizer for agricultural crops in farming practices worldwide and is becoming an extensive reservoir of antimicrobial-resistant bacteria and ARGs. Moreover, bacteria and associated ARGs in livestock manure can be released into the environment and spread onto farmland, which poses significant potential risks to the environment and public health (2).

Linezolid is an effective antimicrobial agent for the treatment of clinical infections caused by MDR Gram-positive bacteria, including methicillin-resistant Staphylococcus aureus (MRSA), vancomycin-resistant enterococci (VRE), and penicillin-resistant Staphylococcus pneumoniae (PRSP) (3). Linezolid has never been approved for use in livestock, however, three acquired linezolid resistance genes (*cfr*, *optrA*, and *poxtA*) have been detected in bacteria of animal origin, arousing public concern. Currently, at least three different groups of acquired resistance genes that confer resistance to linezolid have been reported, including *cfr* and its variants *cfr*(B), *cfr*(C), *cfr*(D), *cfr*(E), *optrA*, and *poxtA* (4–11). More recently, a *poxtA* variant, designated *poxtA2*, was detected in Enterococcus gallinarum (12). The *cfr* gene and its variants code for 23S rRNA methylases, while the *optrA* and *poxtA* genes encode ribosome-protective proteins of the ABC-F family. The *cfr* gene confers resistance to phenicols, lincosamides, oxazolidinones (linezolid but not tedizolid), pleuromutilins, and streptogramin A (the so-called PhLOPSA phenotype). The *optrA* gene, unlike *cfr*, in addition to linezolid and phenicol resistance also confers resistance to tedizolid, a second-generation oxazolidinone. The *poxtA* and *poxtA2* genes also confer linezolid and phenicol resistance. Although initially described to also confer decreased susceptibility to tetracycline, a recent study found no evidence for this (13). The initial identification of partial above-mentioned resistance genes was in clinical samples, such as *cfr*(B)-carrying E. faecalis in United States (5), *cfr*(D)-carrying E. faecium in Australia (7), and *optrA*-carrying E. faecalis and E. faecium in China (9). In addition, *cfr* and *poxtA* genes were also reported in clinical enterococci, and both genes were first reported in E. faecalis, with *cfr* in Thailand (14) and *poxtA* in the United States (15). Moreover, the distribution of *cfr*, *optrA,* and *poxtA* proved that they were affected by the residual concentration of florfenicol in livestock manures, indicating that the use of florfenicol in livestock may lead to the dissemination of these acquired linezolid resistance genes (16).

Previously, porcine E. faecalis isolates harboring *optrA* and *poxtA* genes were identified, and the two resistance genes were located on the same or different plasmids (17, 18). Subsequently, a porcine E. hirae isolate that carried two plasmids with either *poxtA* or *cfr*, respectively, was identified (19). Very recently, a porcine E. gallinarum isolate harboring three linezolid resistance genes was also identified, with one plasmid carrying the *cfr* and *optrA* genes and another the *poxtA* gene (20). Almost simultaneously, a *cfr*(D)- and *poxtA2-*cocarrying plasmid was reported in porcine E. faecalis and E. casseliflavus isolates (21). The co-existence of two linezolid resistance genes, locating on one plasmid or two different plasmids, were identified in clinical samples, such as the *optrA-* and *poxtA*- cocarrying clinical E. faecalis/E. faecium isolates reported in Spain and France (22, 23). In addition, *cfr* and *optrA* cocarrying linezolid-resistant enterococci were reported in Italy and China, locating in same plasmid in E. faecium and two different plasmids in E. faecalis, respectively (24, 25).

In this study, a linezolid-resistant isolate of chicken origin harboring four acquired linezolid resistance genes [*optrA*, *cfr*, *cfr*(D), and *poxtA2*] simultaneously was identified. In addition, their association with mobile genetic elements and their transfer potential were explored.

## RESULTS AND DISCUSSION

### A linezolid-resistant E. faecalis isolate cocarried *optrA*, *cfr*, *cfr*(D), and *poxtA2*.

The linezolid-resistant ST256 E. faecalis QZ076 was identified (MIC, 8 mg/L), which also displayed resistance to florfenicol (MIC, >128 mg/L) and tetracycline (MIC, >128 mg/L) but susceptibility to tedizolid (MIC, 0.5 mg/L). It carried four acquired linezolid-resistant genes, *optrA*, *cfr*, *cfr*(D), and *poxtA2*, simultaneously, with *optrA* being located in the chromosomal DNA and *cfr*, *cfr*(D), and *poxtA2* in plasmids.

The *optrA* gene in E. faecalis QZ076 was located on a Tn*6674*-like transposon, which revealed 99% identity (coverage, 100%) with that in E. faecalis E1731 (accession no. MK737778.1) deposited in GenBank (26, 27), as shown in Fig. S1 in the supplemental material. This transposon represents a member of the Tn*554* family and was integrated into the chromosomal *radC* gene, coding for a DNA repair protein, the preferential integration site of these transposons. It differed from Tn*6674* by a 900-bp sequence deletion in noncoding region. The *optrA* gene codes for an ABC-F protein of 655 amino acids (aa), which differs from the original OptrA protein of plasmid pE349 from E. faecalis at five positions: K3**E**, N12**Y**, G40**D**, I287**K**, and G393**D** (9). This EYDKD variant represents a novel OptrA variant that has not been described before (11).

**FIG 1:**
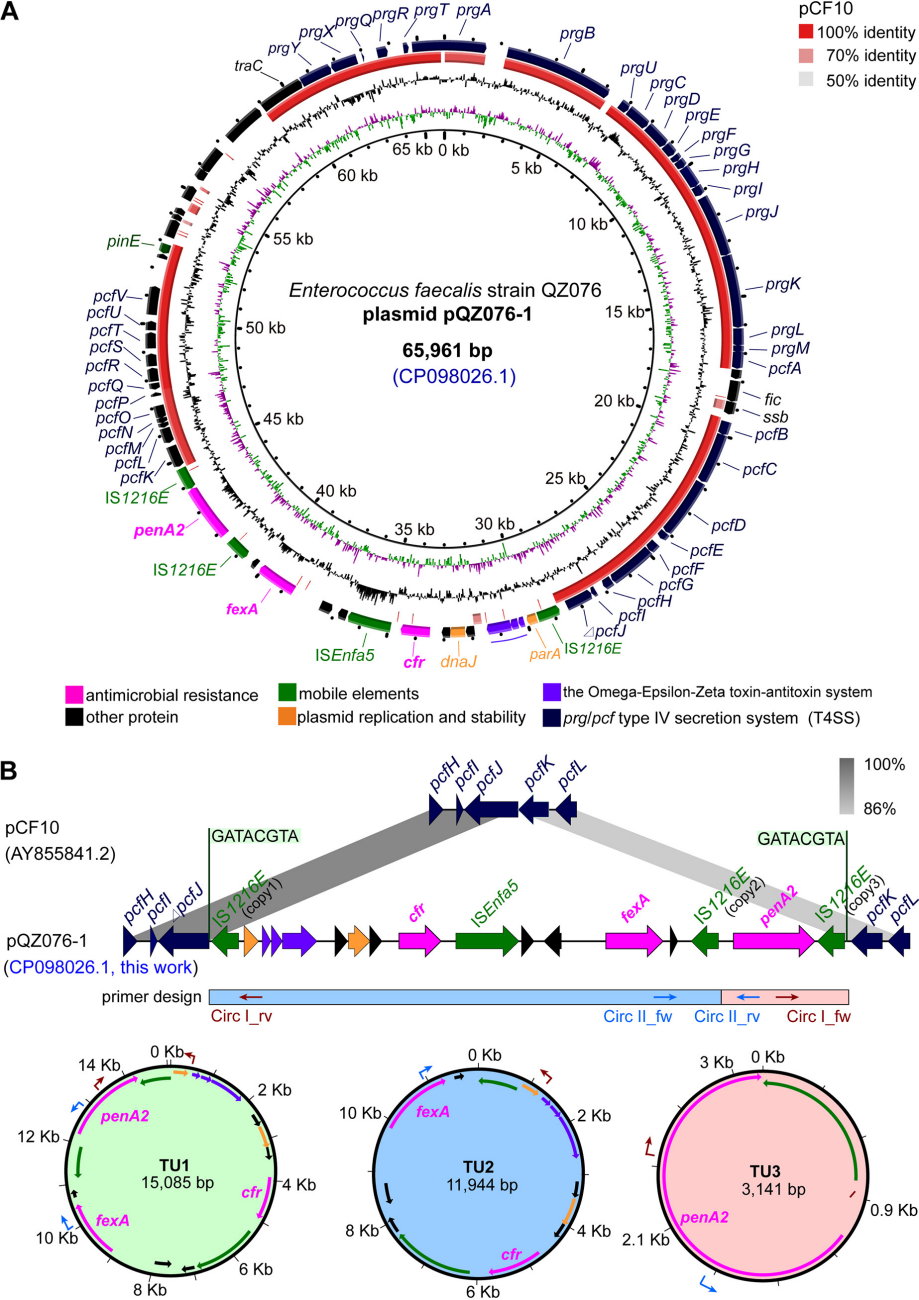
(A) Structural comparison of cfr-carrying plasmids pQZ076-1 (GenBank accession no. CP098026.1) and pCF10 (GenBank accession no. AY855841.2) using BRIG software. Plasmids included in the analysis were as follows: (inner to outer circles) pcf10 and CDS in pQZ076-1. (B) Structural comparison of *cfr*-carrying region on pQZ076-1 with the corresponding part on pcf10 using Easyfig software. Three TUs (TU1, 15,085 bp; TU2, 11,944 bp; TU3, 3,141 bp) formed by IS*1216E*-mediated recombination on this region were indicated.

E. faecalis QZ076 carried four plasmids, designated pQZ076-1 to pQZ076-4, respectively. pQZ076-1 was 65,961 bp in size and harbored the multiresistance gene *cfr*, the penicillin resistance gene *penA2*, and the florfenicol resistance gene *fexA* (Fig. 1A). BLASTN analysis revealed that the backbone of pQZ076-1 differed significantly from that of previously reported *cfr-*carrying plasmids but revealed 99% nucleotide sequence identity (coverage, 61%) with pCF10, the best studied pheromone-responsive conjugative plasmid (28). pQZ076-1 harbored all essential sex pheromone response genes and genes for a type 4 secretion system (T4SS) except that the *pcfJ* gene was truncated (Fig. 1A).

pQZ076-2 was 59,955 bp in size and harbored the tetracycline resistance genes *tet*(M) and *tet*(L), and it revealed approximately 99% nucleotide sequence identity (coverage, 63%) with pS7316 from a linezolid-resistant human E. faecalis isolate (Fig. S2).

pQZ076-3 was 55,876 bp in size and harbored the trimethoprim resistance gene *dfrG*, the neomycin-kanamycin resistance gene *aphA3*, and the macrolide-lincosamide-streptogramin B resistance gene *erm*(B). It revealed about 99% nucleotide sequence identity (coverage, 83%) with pE035 from a linezolid-resistant E. faecalis isolate of porcine origin (Fig. S3) (17).

**FIG 2:**
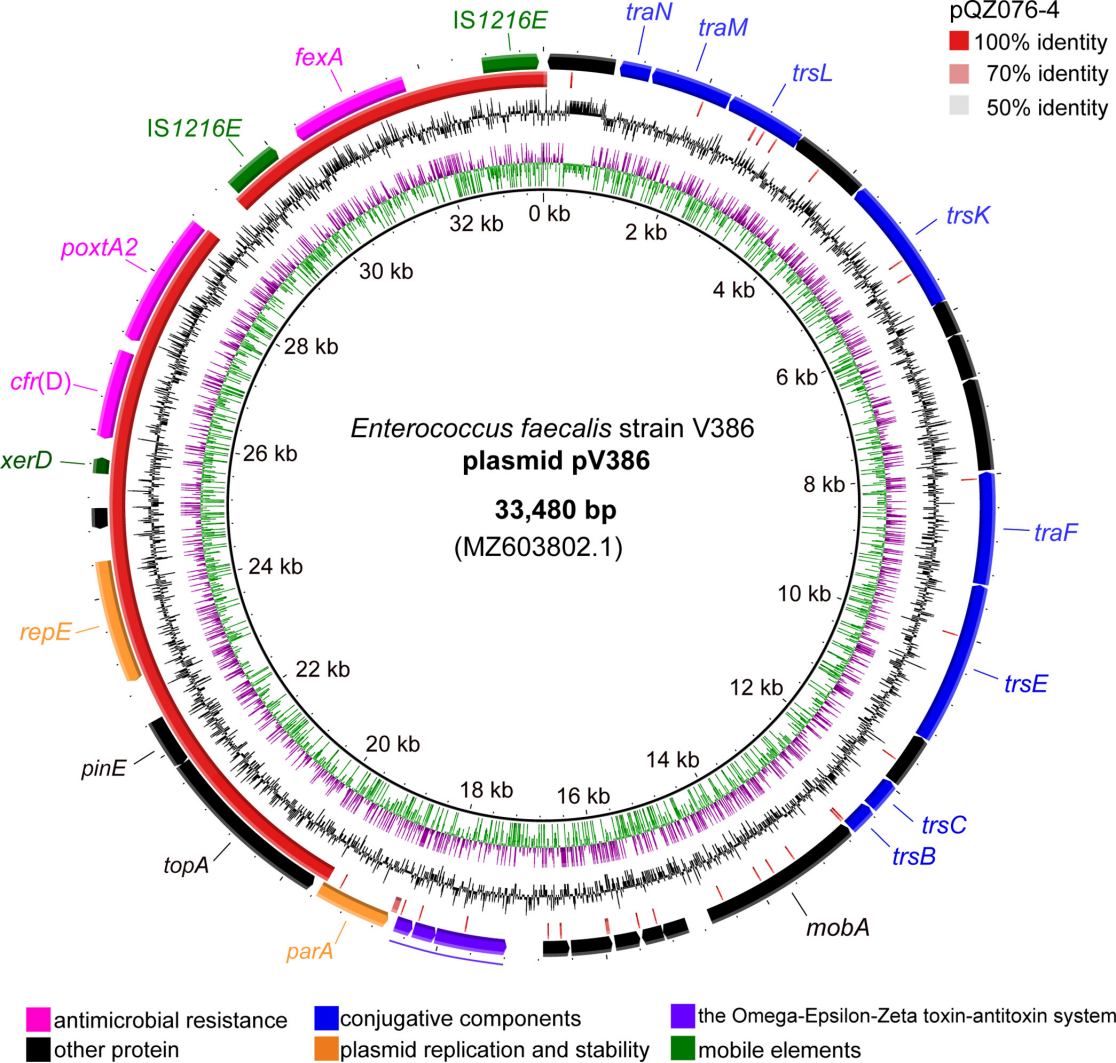
Structural comparison of plasmids pQZ076-4 (GenBank accession no. CP098029.1) and pV386 (GenBank accession no. MZ603802.1) using BRIG software. Plasmids included in the analysis were as follows: (inner to outer circles) pQZ076-4 and CDS in pV386.

pQZ076-4 had a size of 16,397 bp and harbored *fexA*, the multiresistance gene *cfr*(D), and *poxtA2* (Fig. 2). BLASTN analysis revealed that pQZ076-4 shared 98% nucleotide identity (coverage, 82%) with the *cfr*(D)- and *poxtA2*-cocarrying plasmid pV386 from the linezolid-resistant *E. casseliflavus* isolated from porcine manure (25). However, pQZ076-4 lacked the conjugation-related region, compared to the conjugative plasmid pV386.

### Novel *cfr*-embedded pseudocompound transposon Tn*7515* inserted into a pCF10-like pheromone-responsive conjugative plasmid.

The gene *cfr* was flanked by three identical copies of IS*1216E* in the same orientation, one upstream and two downstream of *cfr*. Moreover, the structure bounded by the two terminal IS*1216E* copies (IS*1216E* copy 1 and copy 3) formed a novel pseudocompound transposon, designated Tn*7515*, which was integrated into the *pcfJ* gene of pQZ076-1 and thereby generated 8-bp direct target site duplications (5′-GATACGTA-3′) (Fig. 1B). A similar study also showed that the *cfr* gene had been flanked by two copies of IS*1216E* on the nonconjugative pEF-01 from the bovine E. faecalis strain EF-01, however, no direct repeats were observed in that case at the boundaries of IS*1216E* (29).

To investigate whether the pseudo-compound transposon Tn*7515* on pQZ076-1 was active, a PCR assay was designed and the result indicated a 15,085-bp TU1 formed by recombination between the IS*1216E* copies 1 and 3 (Fig. 1B). In addition, PCR assays were performed to detect whether other TUs can be formed by recombination of IS*1216E* copies 2 and 3, or IS*1216E* copies 1 and 2, respectively, and the results revealed that the corresponding circular intermediates were formed, designated TU2 and TU3, respectively (Fig. 1B). The length of TU2 and TU3 was 11,944 bp, and 3,141 bp, respectively.

### *cfr*(D) and *poxtA2* were located on a broad-host-range Inc18 plasmid.

PlasmidFinder results showed that pQZ076-4 belonged to the broad-host-range Inc18 family of plasmids, which replicates by the theta mechanism. BLASTN analysis revealed that the *rep* gene on pQZ076-4 showed 96.88% nucleotide identity with that on pIP501 in Streptococcus agalactiae, a streptococcal plasmid belonging to the Inc18 family. Inc18 family plasmids can be transferred by conjugation to a wide variety of bacteria, including streptococci, lactococci, staphylococci, and enterococci under *in vitro* conditions (30). The results of mating experiments confirmed that a *vanA*-carrying Inc18-like plasmid could also be transferred from an E. faecalis isolate to a MRSA isolate (31), demonstrating the dissemination potential of Inc18 family plasmids between enterococci and staphylococci.

### Intra- or interspecies transfer of *cfr*(D)- and *poxtA2*-cocarrying pQZ076-4 can occur with the aid of the *cfr*-carrying pheromone responsive conjugative plasmid pQZ076-1.

The *cfr*-carrying pheromone-responsive plasmid pQZ076-1 was successfully transferred by conjugation from the donor strain E. faecalis QZ076 to the recipient strain E. faecalis JH2-2 (ST8), with a frequency of 4.3 × 10^−4^. The corresponding transconjugant, designated E. faecalis QZ076 × JH2-2-TC1 displayed resistance to florfenicol and reduced susceptibility to linezolid (Table S1). This transfer was foreseeable due to the conjugation ability of plasmid pQZ076-1. In addition, cotransfer of the *cfr*(D)- and *poxtA2*-cocarrying plasmid pQZ076-4 via mobilization by the pheromone-responsive conjugative plasmid pQZ076-1 was also observed (Fig. S4).

To investigate whether the *cfr*(D)- and *poxtA2*-cocarrying mobilizable broad-host-range Inc18 pQZ076-4 could transfer from E. faecalis QZ076 to S. aureus, conjugation experiments were performed using MRSA 109 as the recipient. The results indicated that transconjugants were successfully obtained. One of them, designated MRSA QZ076 × 109-TC2, was further investigated. This transconjugant carried *cfr*(D), *poxtA2*, and *fexA* genes, but none of the other resistance genes, suggesting that only the *cfr*(D)- and *poxtA2*-cocarrying plasmid pQZ076-4 was transferred to MRSA 109 (Fig. S4).

Previously, we have reported that a *poxtA*-carrying plasmid could transfer within an E. faecalis population with the aid of a pheromone-responsive conjugative plasmid (18). In this study, we could also show that pheromone-responsive conjugative plasmid was responsible for the interspecies transfer of a nonconjugative broad-host-range Inc18 plasmid from E. faecalis to S. aureus.

## MATERIALS AND METHODS

### Bacterial strains and PCR analysis.

E. faecalis QZ076 was isolated from a fecal sample of chicken origin in Zhejiang Province, China, in 2021. Species assignment and the presence of oxazolidinone resistance genes were tested by PCR. In addition, the presence of circular intermediates was confirmed by PCR using the primers listed in Table S2. All PCR products were subjected to Sanger sequencing.

### Antimicrobial susceptibility testing.

The susceptibility of isolates to linezolid, tedizolid, florfenicol, and tetracycline was tested by standard broth microdilution assays, and the results were interpreted according to the CLSI recommendations (32). E. faecalis ATCC 29212 served as the quality control strain.

### Transfer experiments.

Conjugation experiments were carried out as previously described (33), using E. faecalis JH2-2 (RIF^r^) and MRSA 109 as recipients. As a clinical isolate, MRSA 109 was plasmid-free and resistant to rifampin (34). Transconjugants were selected on brain heart infusion agar supplemented with 32 mg/L rifampicin and 8 mg/L florfenicol. Colonies that grew on these double-selective plates were evaluated for their susceptibility to linezolid, tedizolid, florfenicol, and tetracycline and further confirmed by PCR and multilocus sequence typing (MLST) following harmonized protocols.

### WGS and sequence analysis.

Genomic DNA was extracted using a genomic DNA purification kit (Generay, Shanghai, China). The concentration and purity of the DNA in the samples were detected using a NanoDrop 2000 spectrophotometer (Thermo Fisher, Waltham, MA, USA) and a Qubit 3.0 fluorometer (Invitrogen, USA), respectively. WGS was performed using the Illumina NovaSeq 6000 and the Nanopore sequencing GridION platforms. Sequence assembly was then performed with Unicycler v. 0.4.3. In addition, the NCBI prokaryotic genome annotation pipeline was used for gene annotation. Acquired AMR genes were predicted using ResFinder 4.1 (https://cge.cbs.dtu.dk/services/ResFinder/). The MLST information of the isolates was extracted from assembled genomes. The plasmid sequence was annotated using RAST (http://rast.nmpdr.org/) followed by manual review using BLAST and the plasmid replicon genotype was identified using Plasmid Finder (https://cge.cbs.dtu.dk/services/PlasmidFinder/) (35). Insertion sequence (IS) elements were identified using ISfinder (https://isfinder.biotoul.fr/) (36). The transposon name was assigned by the Tn Registry website curators (https://transposon.lstmed.ac.uk/). Comparative analysis and plasmid maps were generated by BRIG (37).

### Data availability.

The chromosome sequence of E. faecalis strains QZ076 and the plasmid sequences of pQZ076-1, pQZ076-2, pQZ076-3, and pQZ076-4 have been deposited in GenBank under accession numbers CP098025.1, CP098026.1, CP098027.1, CP098028.1, and CP098029.1, respectively.
