# Supplementary material for: Linezolid-Resistant Enterococcus faecalis of Chicken Origin Harbored Chromosome-Borne optrA and Plasmid-Borne cfr, cfr(D), and poxtA2 Genes
Source: Microbiol Spectr. 2023 Mar 30;11(3):e02741-22. doi: 10.1128/spectrum.02741-22 (PMC10269796; doi:10.1128/spectrum.02741-22)
Supplement: Supplemental file 1 — Supplemental material. Download spectrum.02741-22-s0001.pdf, PDF file, 0.6 MB [file spectrum.02741-22-s0001.pdf]

The Supplementary data

Table S1. The antimicrobial susceptibilities of the donor strains, transconjugants and the recipient strains in this study

Strains	MICs (mg/L) [a]			
	FFC	LZD	TZD	TET
Donor strains				
QZ076	>128	8	0.5	>128
Transconjugants				
E. faecalis QZ076×JH2-2-TC1	64	4	0.5	<1
MRSA QZ076×109-TC2	256	2	<0.25	16
Recipient strains				
E. faecalis JH2-2	4	2	0.5	<1
MRSA109	4	2	<0.25	16

[a] FFC, florfenicol; LZD, linezolid; TZD, Tedizolid; TET, tetracycline;

Table S2. PCR primers used in this study

Category	Primer designation	Sequence (5'-3')	Product size (bp)	Reference
TU1	Circ-I-fw	GCACTTGTTTATCCGCAAA	2,653	This study
	Circ-I-rw	GTTGTTCGTCCTGTACCTTC		
TU2	Circ-II-fw	GGTTTATTCCTCGGCCTCA	3,619	This study
	Circ-I-rv	GTTGTTCGTCCTGTACCTTC		
TU3	Circ-I-fw	GCACTTGTTTATCCGCAAA	2,110	This study
	Circ-II-rv	CTTCAACAGAAACACCGAAC		

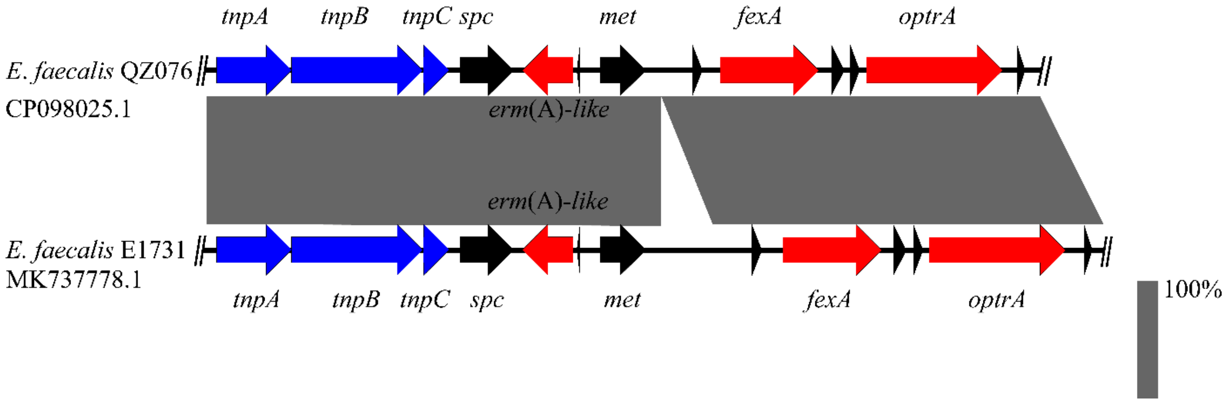

Figure S1. Structure comparison between the genetic context of the *optrA* gene in *E. faecalis* QZ076 and Tn*6674* in *E. faecalis* E1731deposited in GenBank. Antimicrobial resistance genes are shown in red, transposase genes (*tnpA*, *tnpB*, and *tnpC*) in blue, and other genes in black.

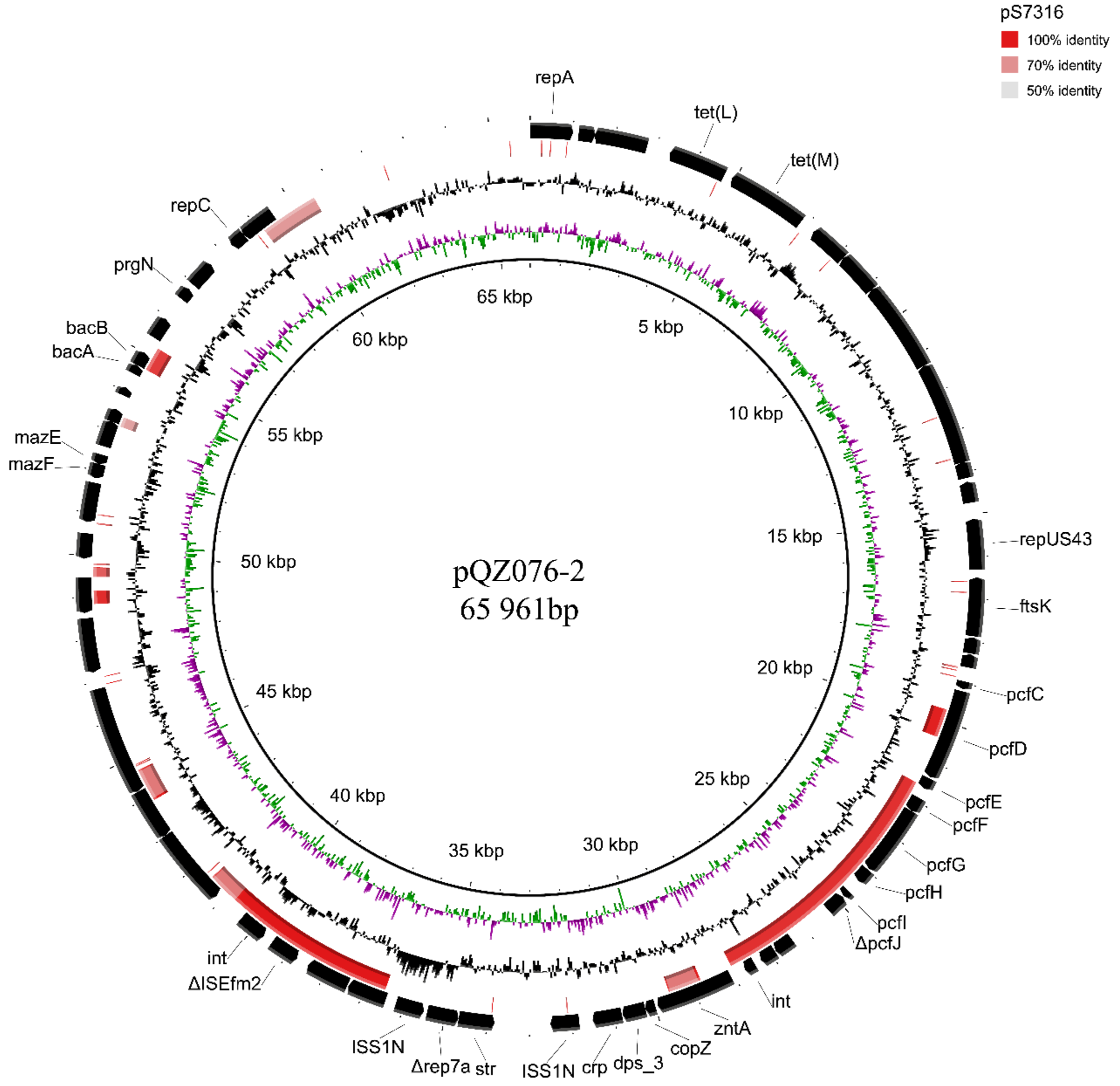

Figure S2. Structural comparison of plasmids pQZ076-2 (GenBank accession no. CP098027.1) and pS7316 (GenBank accession no. LC499744.1) using BRIG software. Plasmids included in the analysis were as follows: (inner to outer circles), pS7316 and CDS in pQZ076-2.

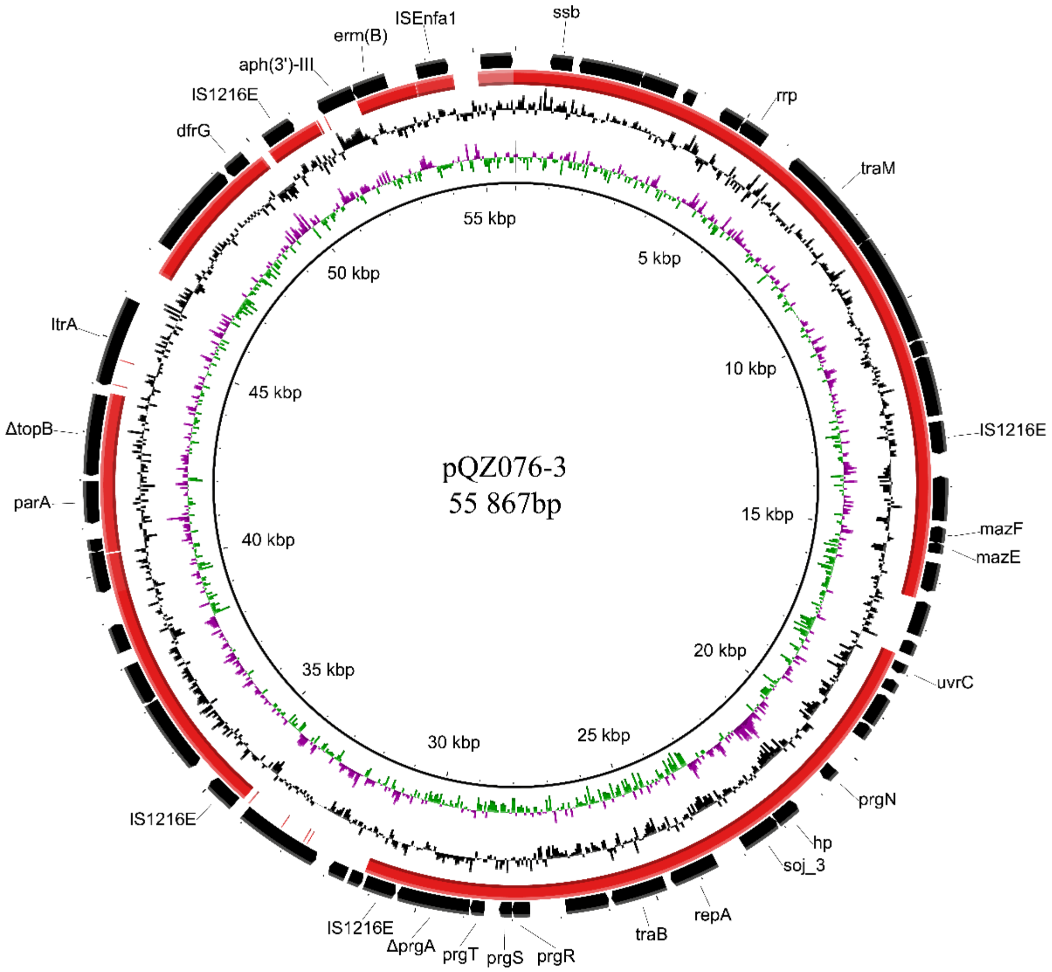

Figure S3. Structural comparison of plasmids pQZ076-3 (GenBank accession no. CP098028.1) and pE035 (GenBank accession no. MK140641.1) using BRIG software. Plasmids included in the analysis were as follows: (inner to outer circles) pE035 and CDS in pQZ076-3.

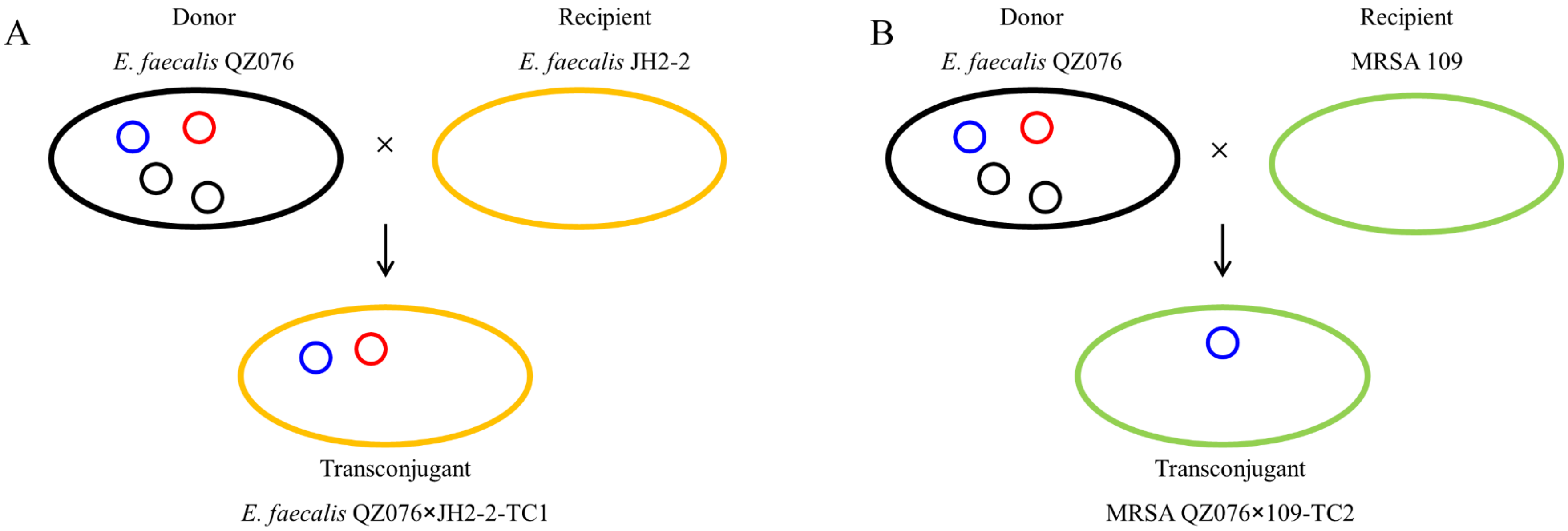

Figure S4. Simplified schematic diagram for transfer experiments. A) Intra species transfer of *cfr*(D)- and *poxtA2*-co-carrying pQZ076-4 and *cfr*-carrying pheromone-responsive conjugative plasmid pQZ076-1. B) Inter species transfer of pQZ076-4. The plasmid pQZ076-4 is shown in blue, pQZ076-1 in red, and the other two plasmids in *E. faecalis* QZ076 in black.
